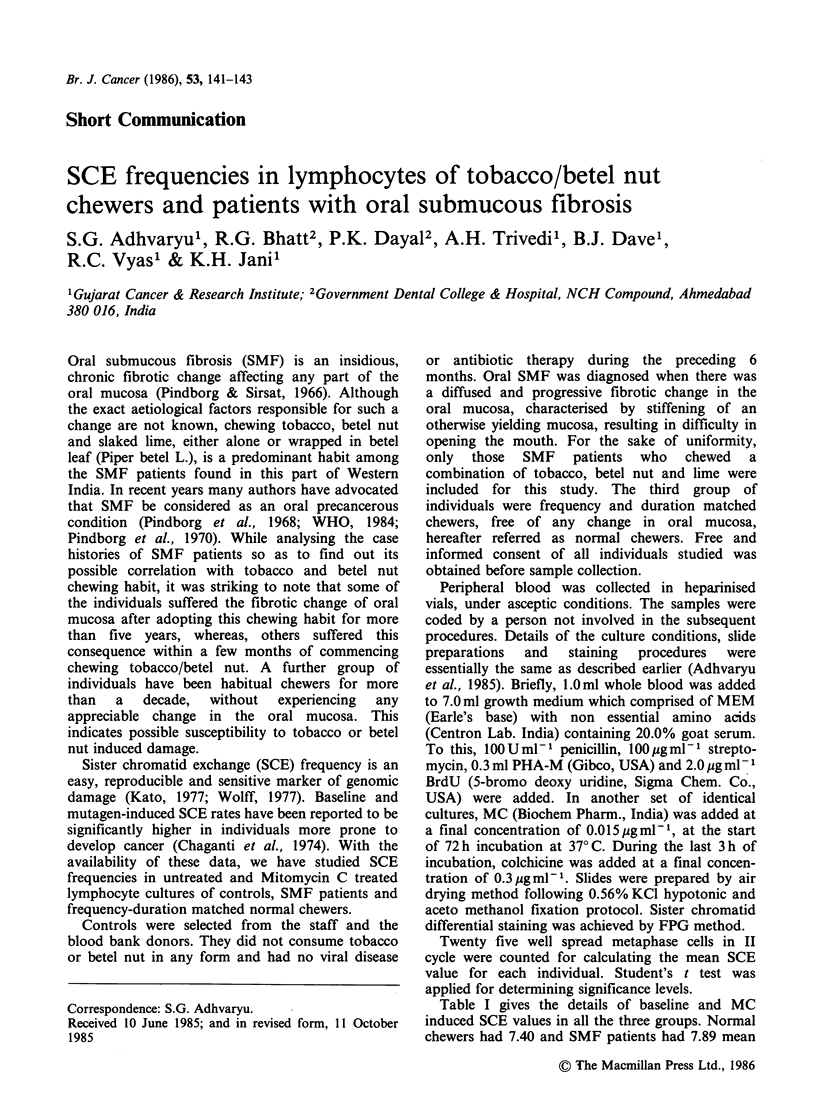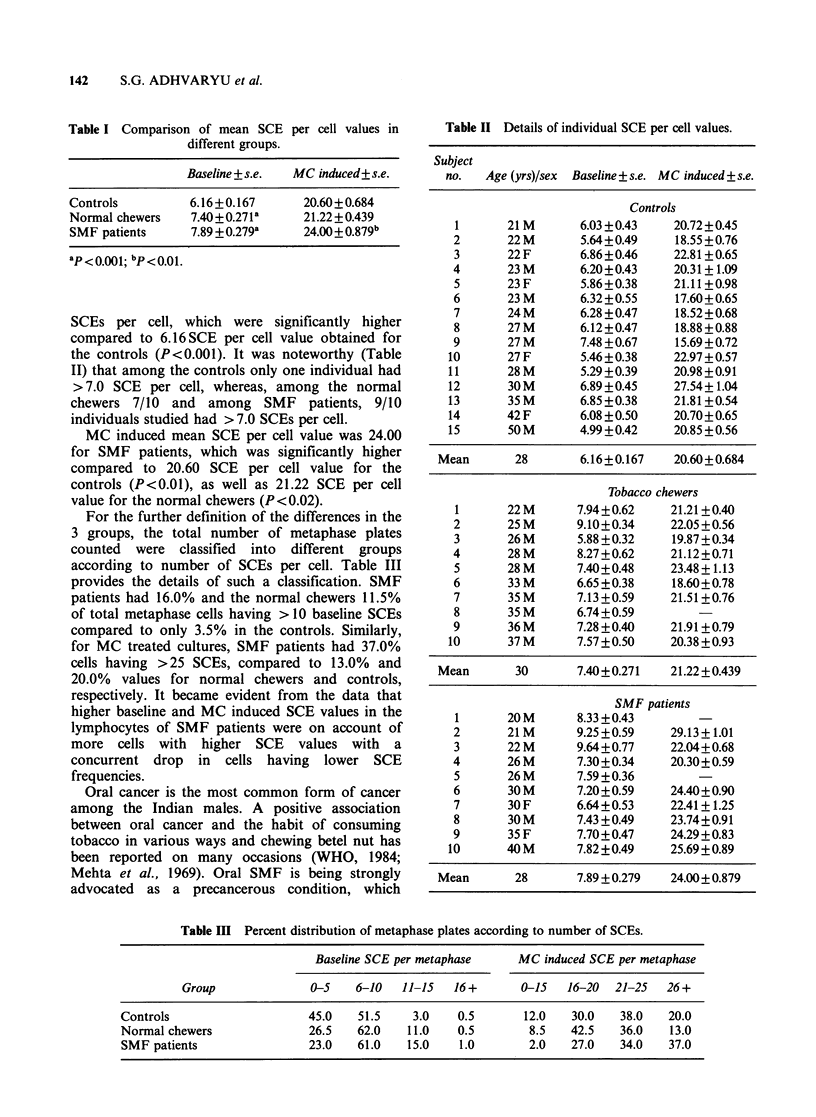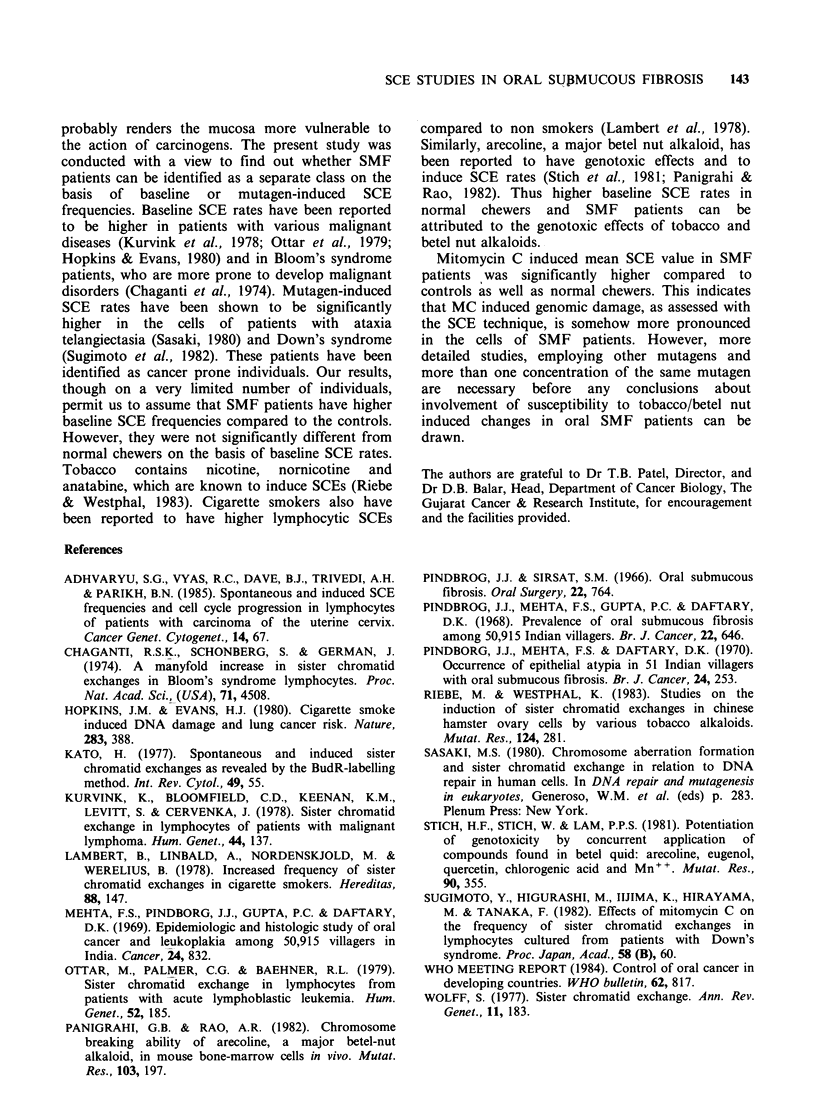# SCE frequencies in lymphocytes of tobacco/betel nut chewers and patients with oral submucous fibrosis.

**DOI:** 10.1038/bjc.1986.20

**Published:** 1986-01

**Authors:** S. G. Adhvaryu, R. G. Bhatt, P. K. Dayal, A. H. Trivedi, B. J. Dave, R. C. Vyas, K. H. Jani


*Br. J. Cancer* (1986), **53**, 141–143

### Short Communication

### SCE frequencies in lymphocytes of tobacco/betel nut chewers and patients with oral submucous fibrosis

S.G. Adhvaryu[1], R.G. Bhatt[2], P.K. Dayal[2], A.H. Trivedi[1], B.J. Dave[1], R.C. Vyas[1] & K.H. Jani[1]

[1]*Gujarat Cancer & Research Institute;* [2]*Government Dental College & Hospital, NCH Compound, Ahmedabad 380 016, India*

Oral submucous fibrosis (SMF) is an insidious, chronic fibrotic change affecting any part of the oral mucosa (Pindborg & Sirsat, 1966). Although the exact aetiological factors responsible for such a change are not known, chewing tobacco, betel nut and slaked lime, either alone or wrapped in betel leaf (Piper betel L.), is a predominant habit among the SMF patients found in this part of Western India. In recent years many authors have advocated that SMF be considered as an oral precancerous condition (Pindborg *et al.,* 1968; WHO, 1984; Pindborg *et al.,* 1970). While analysing the case histories of SMF patients so as to find out its possible correlation with tobacco and betel nut chewing habit, it was striking to note that some of the individuals suffered the fibrotic change of oral mucosa after adopting this chewing habit for more than five years, whereas, others suffered this consequence within a few months of commencing chewing tobacco/betel nut. A further group of individuals have been habitual chewers for more than a decade, without experiencing any appreciable change in the oral mucosa. This indicates possible susceptibility to tobacco or betel nut induced damage.

Sister chromatid exchange (SCE) frequency is an easy, reproducible and sensitive marker of genomic damage (Kato, 1977; Wolff, 1977). Baseline and mutagen-induced SCE rates have been reported to be significantly higher in individuals more prone to develop cancer (Chaganti *et al.,* 1974). With the availability of these data, we have studied SCE frequencies in untreated and Mitomycin C treated lymphocyte cultures of controls, SMF patients and frequency-duration matched normal chewers.

Controls were selected from the staff and the blood bank donors. They did not consume tobacco or betel nut in any form and had no viral disease or antibiotic therapy during the preceding 6 months. Oral SMF was diagnosed when there was a diffused and progressive fibrotic change in the oral mucosa, characterised by stiffening of an otherwise yielding mucosa, resulting in difficulty in opening the mouth. For the sake of uniformity, only those SMF patients who chewed a combination of tobacco, betel nut and lime were included for this study. The third group of individuals were frequency and duration matched chewers, free of any change in oral mucosa, hereafter referred as normal chewers. Free and informed consent of all individuals studied was obtained before sample collection.

Peripheral blood was collected in heparinised vials, under asceptic conditions. The samples were coded by a person not involved in the subsequent procedures. Details of the culture conditions, slide preparations and staining procedures were essentially the same as described earlier (Adhvaryu *et al.,* 1985). Briefly, 1.0 ml whole blood was added to 7.0 ml growth medium which comprised of MEM (Earle's base) with non essential amino acids (Centron Lab. India) containing 20.0% goat serum. To this, $100\,U\,ml^{-1}$ penicillin, $100\,\mu g\,ml^{-1}$ streptomycin, 0.3 ml PHA-M (Gibco, USA) and $2.0\,\mu g\,ml^{-1}$ BrdU (5-bromo deoxy uridine, Sigma Chem. Co., USA) were added. In another set of identical cultures, MC (Biochem Pharm., India) was added at a final concentration of $0.015\,\mu g\,ml^{-1}$, at the start of 72 h incubation at 37° C. During the last 3 h of incubation, colchicine was added at a final concentration of $0.3\,\mu g\,ml^{-1}$. Slides were prepared by air drying method following 0.56% KCl hypotonic and aceto methanol fixation protocol. Sister chromatid differential staining was achieved by FPG method.

Twenty five well spread metaphase cells in II cycle were counted for calculating the mean SCE value for each individual. Student's *t* test was applied for determining significance levels.

Table I gives the details of baseline and MC induced SCE values in all the three groups. Normal chewers had 7.40 and SMF patients had 7.89 mean

Correspondence: S.G. Adhvaryu.
Received 10 June 1985; and in revised form, 11 October 1985

© The Macmillan Press Ltd., 1986

**Table I** Comparison of mean SCE per cell values in different groups.

| | *Baseline* ± *s.e.* | *MC induced* ± *s.e.* |
|---|---|---|
| Controls | 6.16 ± 0.167 | 20.60 ± 0.684 |
| Normal chewers | 7.40 ± 0.271[a] | 21.22 ± 0.439 |
| SMF patients | 7.89 ± 0.279[a] | 24.00 ± 0.879[b] |

[a]$P<0.001$; [b]$P<0.01$.

SCEs per cell, which were significantly higher compared to 6.16 SCE per cell value obtained for the controls ($P<0.001$). It was noteworthy (Table II) that among the controls only one individual had >7.0 SCE per cell, whereas, among the normal chewers 7/10 and among SMF patients, 9/10 individuals studied had >7.0 SCEs per cell.

MC induced mean SCE per cell value was 24.00 for SMF patients, which was significantly higher compared to 20.60 SCE per cell value for the controls ($P<0.01$), as well as 21.22 SCE per cell value for the normal chewers ($P<0.02$).

For the further definition of the differences in the 3 groups, the total number of metaphase plates counted were classified into different groups according to number of SCEs per cell. Table III provides the details of such a classification. SMF patients had 16.0% and the normal chewers 11.5% of total metaphase cells having >10 baseline SCEs compared to only 3.5% in the controls. Similarly, for MC treated cultures, SMF patients had 37.0% cells having >25 SCEs, compared to 13.0% and 20.0% values for normal chewers and controls, respectively. It became evident from the data that higher baseline and MC induced SCE values in the lymphocytes of SMF patients were on account of more cells with higher SCE values with a concurrent drop in cells having lower SCE frequencies.

Oral cancer is the most common form of cancer among the Indian males. A positive association between oral cancer and the habit of consuming tobacco in various ways and chewing betel nut has been reported on many occasions (WHO, 1984; Mehta *et al.*, 1969). Oral SMF is being strongly advocated as a precancerous condition, which

**Table II** Details of individual SCE per cell values.

| *Subject no.* | *Age (yrs)/sex* | *Baseline* ± *s.e.* | *MC induced* ± *s.e.* |
|---|---|---|---|
| | | *Controls* | |
| 1 | 21 M | 6.03 ± 0.43 | 20.72 ± 0.45 |
| 2 | 22 M | 5.64 ± 0.49 | 18.55 ± 0.76 |
| 3 | 22 F | 6.86 ± 0.46 | 22.81 ± 0.65 |
| 4 | 23 M | 6.20 ± 0.43 | 20.31 ± 1.09 |
| 5 | 23 F | 5.86 ± 0.38 | 21.11 ± 0.98 |
| 6 | 23 M | 6.32 ± 0.55 | 17.60 ± 0.65 |
| 7 | 24 M | 6.28 ± 0.47 | 18.52 ± 0.68 |
| 8 | 27 M | 6.12 ± 0.47 | 18.88 ± 0.88 |
| 9 | 27 M | 7.48 ± 0.67 | 15.69 ± 0.72 |
| 10 | 27 F | 5.46 ± 0.38 | 22.97 ± 0.57 |
| 11 | 28 M | 5.29 ± 0.39 | 20.98 ± 0.91 |
| 12 | 30 M | 6.89 ± 0.45 | 27.54 ± 1.04 |
| 13 | 35 M | 6.85 ± 0.38 | 21.81 ± 0.54 |
| 14 | 42 F | 6.08 ± 0.50 | 20.70 ± 0.65 |
| 15 | 50 M | 4.99 ± 0.42 | 20.85 ± 0.56 |
| Mean | 28 | 6.16 ± 0.167 | 20.60 ± 0.684 |
| | | *Tobacco chewers* | |
| 1 | 22 M | 7.94 ± 0.62 | 21.21 ± 0.40 |
| 2 | 25 M | 9.10 ± 0.34 | 22.05 ± 0.56 |
| 3 | 26 M | 5.88 ± 0.32 | 19.87 ± 0.34 |
| 4 | 28 M | 8.27 ± 0.62 | 21.12 ± 0.71 |
| 5 | 28 M | 7.40 ± 0.48 | 23.48 ± 1.13 |
| 6 | 33 M | 6.65 ± 0.38 | 18.60 ± 0.78 |
| 7 | 35 M | 7.13 ± 0.59 | 21.51 ± 0.76 |
| 8 | 35 M | 6.74 ± 0.59 | — |
| 9 | 36 M | 7.28 ± 0.40 | 21.91 ± 0.79 |
| 10 | 37 M | 7.57 ± 0.50 | 20.38 ± 0.93 |
| Mean | 30 | 7.40 ± 0.271 | 21.22 ± 0.439 |
| | | *SMF patients* | |
| 1 | 20 M | 8.33 ± 0.43 | — |
| 2 | 21 M | 9.25 ± 0.59 | 29.13 ± 1.01 |
| 3 | 22 M | 9.64 ± 0.77 | 22.04 ± 0.68 |
| 4 | 26 M | 7.30 ± 0.34 | 20.30 ± 0.59 |
| 5 | 26 M | 7.59 ± 0.36 | — |
| 6 | 30 M | 7.20 ± 0.59 | 24.40 ± 0.90 |
| 7 | 30 F | 6.64 ± 0.53 | 22.41 ± 1.25 |
| 8 | 30 M | 7.43 ± 0.49 | 23.74 ± 0.91 |
| 9 | 35 F | 7.70 ± 0.47 | 24.29 ± 0.83 |
| 10 | 40 M | 7.82 ± 0.49 | 25.69 ± 0.89 |
| Mean | 28 | 7.89 ± 0.279 | 24.00 ± 0.879 |

**Table III** Percent distribution of metaphase plates according to number of SCEs.

| | *Baseline SCE per metaphase* | | | | *MC induced SCE per metaphase* | | | |
|---|---|---|---|---|---|---|---|---|
| *Group* | *0–5* | *6–10* | *11–15* | *16+* | *0–15* | *16–20* | *21–25* | *26+* |
| Controls | 45.0 | 51.5 | 3.0 | 0.5 | 12.0 | 30.0 | 38.0 | 20.0 |
| Normal chewers | 26.5 | 62.0 | 11.0 | 0.5 | 8.5 | 42.5 | 36.0 | 13.0 |
| SMF patients | 23.0 | 61.0 | 15.0 | 1.0 | 2.0 | 27.0 | 34.0 | 37.0 |

probably renders the mucosa more vulnerable to the action of carcinogens. The present study was conducted with a view to find out whether SMF patients can be identified as a separate class on the basis of baseline or mutagen-induced SCE frequencies. Baseline SCE rates have been reported to be higher in patients with various malignant diseases (Kurvink *et al.*, 1978; Ottar *et al.*, 1979; Hopkins & Evans, 1980) and in Bloom's syndrome patients, who are more prone to develop malignant disorders (Chaganti *et al.*, 1974). Mutagen-induced SCE rates have been shown to be significantly higher in the cells of patients with ataxia telangiectasia (Sasaki, 1980) and Down's syndrome (Sugimoto *et al.*, 1982). These patients have been identified as cancer prone individuals. Our results, though on a very limited number of individuals, permit us to assume that SMF patients have higher baseline SCE frequencies compared to the controls. However, they were not significantly different from normal chewers on the basis of baseline SCE rates. Tobacco contains nicotine, nornicotine and anatabine, which are known to induce SCEs (Riebe & Westphal, 1983). Cigarette smokers also have been reported to have higher lymphocytic SCEs compared to non smokers (Lambert *et al.*, 1978). Similarly, arecoline, a major betel nut alkaloid, has been reported to have genotoxic effects and to induce SCE rates (Stich *et al.*, 1981; Panigrahi & Rao, 1982). Thus higher baseline SCE rates in normal chewers and SMF patients can be attributed to the genotoxic effects of tobacco and betel nut alkaloids.

Mitomycin C induced mean SCE value in SMF patients was significantly higher compared to controls as well as normal chewers. This indicates that MC induced genomic damage, as assessed with the SCE technique, is somehow more pronounced in the cells of SMF patients. However, more detailed studies, employing other mutagens and more than one concentration of the same mutagen are necessary before any conclusions about involvement of susceptibility to tobacco/betel nut induced changes in oral SMF patients can be drawn.

The authors are grateful to Dr T.B. Patel, Director, and Dr D.B. Balar, Head, Department of Cancer Biology, The Gujarat Cancer & Research Institute, for encouragement and the facilities provided.

### References

ADHVARYU, S.G., VYAS, R.C., DAVE, B.J., TRIVEDI, A.H. & PARIKH, B.N. (1985). Spontaneous and induced SCE frequencies and cell cycle progression in lymphocytes of patients with carcinoma of the uterine cervix. *Cancer Genet. Cytogenet.*, **14**, 67.

CHAGANTI, R.S.K., SCHONBERG, S. & GERMAN, J. (1974). A manyfold increase in sister chromatid exchanges in Bloom's syndrome lymphocytes. *Proc. Nat. Acad. Sci.*, (*USA*), **71**, 4508.

HOPKINS, J.M. & EVANS, H.J. (1980). Cigarette smoke induced DNA damage and lung cancer risk. *Nature*, **283**, 388.

KATO, H. (1977). Spontaneous and induced sister chromatid exchanges as revealed by the BudR-labelling method. *Int. Rev. Cytol.*, **49**, 55.

KURVINK, K., BLOOMFIELD, C.D., KEENAN, K.M., LEVITT, S. & CERVENKA, J. (1978). Sister chromatid exchange in lymphocytes of patients with malignant lymphoma. *Hum. Genet.*, **44**, 137.

LAMBERT, B., LINBALD, A., NORDENSKJOLD, M. & WERELIUS, B. (1978). Increased frequency of sister chromatid exchanges in cigarette smokers. *Hereditas*, **88**, 147.

MEHTA, F.S., PINDBORG, J.J., GUPTA, P.C. & DAFTARY, D.K. (1969). Epidemiologic and histologic study of oral cancer and leukoplakia among 50,915 villagers in India. *Cancer*, **24**, 832.

OTTAR, M., PALMER, C.G. & BAEHNER, R.L. (1979). Sister chromatid exchange in lymphocytes from patients with acute lymphoblastic leukemia. *Hum. Genet.*, **52**, 185.

PANIGRAHI, G.B. & RAO, A.R. (1982). Chromosome breaking ability of arecoline, a major betel-nut alkaloid, in mouse bone-marrow cells *in vivo*. *Mutat. Res.*, **103**, 197.

PINDBROG, J.J. & SIRSAT, S.M. (1966). Oral submucous fibrosis. *Oral Surgery*, **22**, 764.

PINDBROG, J.J., MEHTA, F.S., GUPTA, P.C. & DAFTARY, D.K. (1968). Prevalence of oral submucous fibrosis among 50,915 Indian villagers. *Br. J. Cancer*, **22**, 646.

PINDBORG, J.J., MEHTA, F.S. & DAFTARY, D.K. (1970). Occurrence of epithelial atypia in 51 Indian villagers with oral submucous fibrosis. *Br. J. Cancer*, **24**, 253.

RIEBE, M. & WESTPHAL, K. (1983). Studies on the induction of sister chromatid exchanges in chinese hamster ovary cells by various tobacco alkaloids. *Mutat. Res.*, **124**, 281.

SASAKI, M.S. (1980). Chromosome aberration formation and sister chromatid exchange in relation to DNA repair in human cells. In *DNA repair and mutagenesis in eukaryotes*, Generoso, W.M. *et al.* (eds) p. 283. Plenum Press: New York.

STICH, H.F., STICH, W. & LAM, P.P.S. (1981). Potentiation of genotoxicity by concurrent application of compounds found in betel quid: arecoline, eugenol, quercetin, chlorogenic acid and $Mn^{++}$. *Mutat. Res.*, **90**, 355.

SUGIMOTO, Y., HIGURASHI, M., IIJIMA, K., HIRAYAMA, M. & TANAKA, F. (1982). Effects of mitomycin C on the frequency of sister chromatid exchanges in lymphocytes cultured from patients with Down's syndrome. *Proc. Japan, Acad.*, **58 (B)**, 60.

WHO MEETING REPORT (1984). Control of oral cancer in developing countries. *WHO bulletin*, **62**, 817.

WOLFF, S. (1977). Sister chromatid exchange. *Ann. Rev. Genet.*, **11**, 183.